# Recommendations for dealing with waste contaminated with Ebola virus: a Hazard Analysis of Critical Control Points approach

**DOI:** 10.2471/BLT.15.163931

**Published:** 2016-03-03

**Authors:** Kelly L Edmunds, Samira Abd Elrahman, Diana J Bell, Julii Brainard, Samir Dervisevic, Tsimbiri P Fedha, Roger Few, Guy Howard, Iain Lake, Peter Maes, Joseph Matofari, Harvey Minnigh, Ahmed A Mohamedani, Maggie Montgomery, Sarah Morter, Edward Muchiri, Lutendo S Mudau, Benedict M Mutua, Julius M Ndambuki, Katherine Pond, Mark D Sobsey, Mike van der Es, Mark Zeitoun, Paul R Hunter

**Affiliations:** a Centre for Ecology, Evolution and Conservation, University of East Anglia, Norwich, England.; b Department of Family and Community Medicine, University of Gezira, Gezira, Sudan.; c Norwich Medical School, University of East Anglia, Norwich, NR4 7TJ, England.; d Reproductive Health Department, Egerton University, Egerton, Kenya.; e School of International Development, University of East Anglia, Norwich, England.; f Department for International Development, East Kilbride, Scotland.; g School of Environmental Sciences, University of East Anglia, Norwich, England.; h Médecins Sans Frontières, Bruxelles, Belgium.; i Department of Dairy, Food Science and Technology, Egerton University, Egerton, Kenya.; j Centro de Educación, Conservación, e Interpretación Ambiental, Inter American University of Puerto Rico, San Germán, Puerto Rico.; k Department of Pathology, University of Gezira, Gezira, Sudan.; l Water, Sanitation, Hygiene and Health Unit, World Health Organization, Geneva, Switzerland.; m Norfolk and Norwich University Hospitals NHS Foundation Trust, Norwich, England.; n Faculty of Engineering and Technology, Egerton University, Egerton, Kenya.; o Tshwane University of Technology, Pretoria, South Africa.; p Department of Civil and Environmental Engineering, University of Surrey, Guildford, England.; q Gillings School of Global Public Health, University of North Carolina, Chapel Hill, United States of America.; r Water Security Research Centre, University of East Anglia, Norwich, England.

## Abstract

**Objective:**

To assess, within communities experiencing Ebola virus outbreaks, the risks associated with the disposal of human waste and to generate recommendations for mitigating such risks.

**Methods:**

A team with expertise in the Hazard Analysis of Critical Control Points framework identified waste products from the care of individuals with Ebola virus disease and constructed, tested and confirmed flow diagrams showing the creation of such products. After listing potential hazards associated with each step in each flow diagram, the team conducted a hazard analysis, determined critical control points and made recommendations to mitigate the transmission risks at each control point.

**Findings:**

The collection, transportation, cleaning and shared use of blood-soiled fomites and the shared use of latrines contaminated with blood or bloodied faeces appeared to be associated with particularly high levels of risk of Ebola virus transmission. More moderate levels of risk were associated with the collection and transportation of material contaminated with bodily fluids other than blood, shared use of latrines soiled with such fluids, the cleaning and shared use of fomites soiled with such fluids, and the contamination of the environment during the collection and transportation of blood-contaminated waste.

**Conclusion:**

The risk of the waste-related transmission of Ebola virus could be reduced by the use of full personal protective equipment, appropriate hand hygiene and an appropriate disinfectant after careful cleaning. Use of the Hazard Analysis of Critical Control Points framework could facilitate rapid responses to outbreaks of emerging infectious disease.

## Résumé

**Objectif:**

Évaluer, au sein des communautés touchées par des flambées de maladie à virus Ebola, les risques liés à l'élimination des déchets et excreta humains et formuler des recommandations pour limiter ces risques.

**Méthodes:**

Une équipe expérimentée dans l'application de la méthode d'analyse des risques et maîtrise des points critiques (HACCP) a identifié les déchets issus des activités de soin prodiguées aux personnes infectées par le virus Ebola et a élaboré, testé et confirmé plusieurs diagrammes des opérations illustrant la génération de ces déchets. Après avoir répertorié les risques potentiels associés à chaque étape de chaque diagramme, l'équipe a mené une analyse des risques, identifié les points critiques pour leur maîtrise et préparé des recommandations pour réduire les risques de transmission à chaque point de maîtrise.

**Résultats:**

La collecte, le transport, le nettoyage et l'utilisation partagée de fomites maculés de sang ainsi que l'utilisation partagée de latrines souillées par du sang ou des fèces contenant du sang semblent être associés à des niveaux de risque de transmission du virus Ebola particulièrement élevés. Des niveaux de risque plus modérés sont associés à la collecte et au transport d'équipements souillés par des fluides corporels autres que le sang, à l'utilisation partagé de latrines souillées par ce type de fluides, au nettoyage et à l'utilisation partagée de fomites souillés par ce type de fluides ainsi qu’à la contamination de l'environnement pendant la collecte et le transport de déchets tachés de sang.

**Conclusion:**

Le risque de transmission du virus Ebola par des déchets pourrait être réduit par l'utilisation d'équipements de protection individuelle complets, par une hygiène des mains appropriée et par l'application d'un désinfectant adapté après un nettoyage scrupuleux. L'utilisation de la méthode d'analyse des risques et maîtrise des points critiques (HACCP) pourrait aider à réagir rapidement en cas de flambée de maladies infectieuses émergentes.

## Resumen

**Objetivo:**

Evaluar, dentro de las comunidades que han sufrido brotes del virus del Ebola, los riesgos relacionados con la eliminación de los desechos humanos y redactar recomendaciones para mitigarlos.

**Métodos:**

Un equipo con experiencia en el análisis de peligros en puntos críticos de control identificó productos de desecho generados por el cuidado de individuos infectados con el virus del Ebola y construyó, probó y confirmó diagramas de flujo que mostraban la creación de dichos productos. Tras redactar una lista de los potenciales peligros relacionados con cada paso de cada diagrama de flujo, el equipo realizó un análisis de peligros, determinó los puntos críticos de control y creó recomendaciones para mitigar los riesgos de contagio en todos los puntos de control.

**Resultados:**

La recolección, el transporte, la limpieza y el uso común de fómites contaminados con sangre y el uso común de letrinas contaminadas con sangre o heces con sangre parecían estar relacionados con riesgos especialmente elevados de contagio del virus del Ebola. Unos niveles de riesgo más moderados se relacionaron con la recolección y el transporte de material contaminado con fluidos corporales distintos a la sangre, el uso común de letrinas sucias con dichos fluidos, la limpieza y el uso de fómites contaminados con dichos fluidos y la contaminación del entorno durante la recolección y el transporte de desperdicios impregnados en sangre.

**Conclusión:**

El riesgo de contagio del virus del Ébola en relación con los desechos podría verse reducido mediante el uso de un equipo de protección individual completo, una higiene de las manos adecuada y un desinfectante apropiado tras una limpieza exhaustiva. El uso del análisis de peligros en puntos críticos de control podría proporcionar una rápida respuesta ante brotes de enfermedades contagiosas emergentes.

## ملخص

**الغرض:**

تقييم المخاطر المرتبطة بالتخلص من النفايات البشرية ووضع توصيات للتخفيف من مثل هذه المخاطر داخل المجتمعات التي تعاني من حالات تفشي فيروس الإيبولا.

**الأسلوب:**

قام فريق من المتمرسين في التعامل مع إطار العمل الخاص بتحليل المخاطر في نقاط التحكم الحرجة بالإشارة إلى النفايات الناتجة عن الرعاية الصحية للمصابين بمرض فيروس الإيبولا باعتبارها مصدرًا للخطر، وقاموا برسم مخططات تدفق توضح نشوء هذه النواتج الخطرة، كما قاموا باختبار تلك المخططات والتأكيد عليها. وبعد سرد المخاطر المحتملة المرتبطة بكل خطوة في كل مخطط من مخططات التدفق، قام الفريق بإجراء تحليل المخاطر، وتحديد نقاط التحكم الحرجة، ووضع التوصيات التي من شأنها التخفيف من مخاطر العدوى في كل نقطة من نقاط التحكم.

**النتائج:**

يبدو أن عمليات جمع الأدوات المعدية المتسخة بالدم ونقلها وتنظيفها والاستخدام المشترك لها، وكذلك الاستخدام المشترك للمراحيض الملوثة بالدم أو البراز المدمى ترتبط بحدوث ارتفاع ملموس في مستويات الخطر لانتقال فيروس الإيبولا. وعلى الجانب الآخر، كانت المستويات الأكثر اعتدالاً من المخاطر قد ارتبطت بعمليات جمع ونقل المواد الملوثة بسوائل الجسم الأخرى غير الدم، والاستخدام المشترك للمراحيض المتسخة بمثل هذه السوائل، وعمليات التنظيف والاستخدام المشترك للأدوات المعدية المتسخة بمثل هذه السوائل، وتلوث البيئة أثناء جمع ونقل النفايات الملوثة بالدم.

**الاستنتاج:**

يمكن خفض خطر انتقال فيروس الإيبولا المتعلق بالنفايات عن طريق استخدام معدات الحماية الشخصية الكاملة، والاهتمام بالنظافة المناسبة لليدين، ووضع مطهر مناسب بعد التنظيف بعناية. ولذلك، فمن الممكن أن يقود الاعتماد على إطار العمل الخاص بتحليل المخاطر في نقاط التحكم الحرجة إلى تيسير سبل الاستجابة السريعة لحالات تفشي الأمراض المعدية الناشئة.

## 摘要

**目的:**

旨在评估埃博拉病毒爆发社区内，与人类废弃物处理相关的风险，并给出降低此类风险的建议。

**方法:**

具备关键控制点框架危害分析专业知识的专家团队，确定了护理埃博拉病毒患者产生的废弃物，并且构建出此类废弃物产生的流程图，同时对该流程图进行测试和确认。 列出与各流程图每一步骤相关的潜在危害之后，该团队展开危害分析，确定了关键控制点并给出了降低各控制点传播风险的建议。

**结果:**

收集、运输、清洁和共用被血液污染的传染物，共用被血液污染的厕所或血迹斑斑的粪便疑似与极高的埃博拉病毒传播风险尤为相关。 收集和运输体液而非血液污染的物质，共用被此类体液污染的厕所，清洁和共用此类体液污染的传染物，以及收集和运输血污染废弃物期间的环境污染与较低风险水平相关。

**结论:**

可通过使用全方位个人防护装备、仔细清洁后正确清洁手部和使用消毒剂，降低与废弃物相关的埃博拉病毒传播风险。 使用关键控制点框架危害分析可促进对新兴传染病疫情爆发的快速响应。

## Резюме

**Цель:**

Провести оценку рисков в связи с утилизацией человеческих отходов в общинах, страдающих от вспышек эпидемии лихорадки Эбола, и составить рекомендации для снижения таких рисков.

**Методы:**

Группа специалистов, обладающих опытом в анализе риска на основе критических контрольных показателей, определила отходы, которые образуются в результате ухода за заразившимися вирусом Эбола, спроектировала и протестировала блок-схемы, иллюстрирующие образование таких отходов, и подтвердила их правильность. Перечислив потенциальные риски, сопряженные с каждым этапом каждой блок-схемы, группа специалистов осуществила анализ риска, определила критические контрольные показатели и составила рекомендации для снижения риска передачи по каждому контрольному показателю.

**Результаты:**

Было определено, что сбор, транспортировка, очистка и коллективное использование загрязненных кровью фомитов и коллективное использование уборных, загрязненных кровью или калом с кровью, ассоциируются с чрезвычайно высоким риском передачи вируса Эбола. Менее высокий уровень риска был связан со сбором и транспортировкой материала, загрязненного биологическими жидкостями, отличными от крови, совместным использованием уборных, содержащих такие жидкости, очисткой и совместным использованием фомитов, загрязненных такими жидкостями, а также с загрязнением окружающей среды при сборе и транспортировке отходов, загрязненных кровью.

**Вывод:**

Риск передачи вируса Эбола, присутствующего в отходах, может быть снижен путем использования полного комплекта средств индивидуальной защиты, следования правилам надлежащей гигиены рук и применения подходящего дезинфицирующего средства после тщательной очистки. Проведение анализа риска на основе критических контрольных показателей могло бы способствовать быстрому реагированию на вспышки эпидемии нового инфекционного заболевания.

## Introduction

Emerging infectious diseases present threats to global health.^1^ The 2014–2016 Ebola virus disease outbreak in western Africa – caused by the Democratic Republic of the Congo strain – caught local and global health-care communities unaware and unprepared. By 2 September 2015, the outbreak had been associated with at least 28 073 confirmed, probable or suspected cases – reported in 10 countries – and more than 11 290 deaths.^2^ There were extraordinary challenges for the health-care systems and local governments involved in the response to the outbreak – and many of those challenges will persist long after the outbreak has passed.^3^ One such challenge was the safe disposal of potentially infected faecal and health-care waste – especially in the overcrowded urban communities where many of the cases occurred. It has been estimated that a patient in a bed within an African centre for Ebola treatment produced up to 300 litres of liquid waste and excreta per day.^4^ Every one of those litres could have been contaminated with Ebola virus^5^ and needed to be disposed of in such a way as to minimize the risks of transmission.^6^ The safe disposal of waste that could harbour Ebola virus is particularly challenging when that waste has been produced beyond any formal health-care setting. In the three countries most affected by the recent outbreak – i.e. Guinea, Liberia and Sierra Leone – severe shortages of water and sanitation services in health-care facilities and the affected communities often complicated the safe disposal of waste. According to the most recent data, nearly one third of health-care facilities in Guinea, Liberia and Sierra Leone have no piped water, 22% have no improved sanitation and 40% have no system to manage health-care waste.^7^ The corresponding percentages for households within the same countries are probably higher.^8^

Most transmission of Ebola virus probably results from direct contact with the bodily fluids of people who are in the latter stages of Ebola virus disease.^9,10^ The survival time of the virus outside its human host is relatively short but – given the size and location of the recent outbreak and the frequent lack of disposal systems – we cannot exclude the possibility that, in some instances, people became infected indirectly, after coming into contact with contaminated waste.

In the present study, we used the Hazard Analysis of Critical Control Points framework to generate recommendations for mitigating the risks posed by virus-contaminated waste within health-care facilities and communities experiencing outbreaks of Ebola virus disease. Although this framework was originally developed for food production systems, it has been successfully adapted to manage and mitigate the risks associated with drinking water.^11,12^ It is increasingly being used to reduce the risks related to emerging infectious diseases and other health threats.^13^ Since its use is both low-technology and relatively cheap, the framework may be particularly useful in addressing the risks associated with emerging infectious disease in areas where the capacity of existing health-care systems is insufficient to cope with the impact of the disease. The framework’s methods encourage the use of interdisciplinary expertise while enabling the rapid generation of evidence-based recommendations. It therefore offers the potential to manage risks when the quick control of an outbreak is essential even though – as with the survival of Ebola virus in sewage and other waste^14^ – the relevant research data are incomplete or can only be inferred. 

In our analyses, we defined waste as both human waste – e.g. faeces and urine – and the fomites generated by health-care activities. We identified those behaviours and practices linked to waste collection and disposal that are likely to present risks of direct or indirect transmission of Ebola virus between humans. We then evaluated the potential of assessments based on the Hazard Analysis of Critical Control Points framework as a response tool during outbreaks of emerging infectious disease.

## Methods

In conducting our Hazard Analysis of Critical Control Points analysis, we adapted the guidance contained within an annex to the Codex Alimentarius Commission’s *General Principles of Food Hygiene*.^15^ Although this guidance refers to 12 steps in the analysis, we ignored some of the later steps in the process because we would not ultimately be responsible for implementing the recommended control measures or for establishing the subsequent on-the-ground monitoring. We used a seven-step process – similar to that used for highly pathogenic avian influenza.^13^ The seven steps were: (i) assemble a team with appropriate expertise in Hazard Analysis of Critical Control Points analysis; (ii) identify waste products within the system of care of cases of Ebola virus disease; (iii) construct flow diagrams illustrating the system of care; (iv) test and confirm the accuracy of each flow diagram; (v) list potential hazards associated with each step in each flow diagram and conduct a hazard analysis; (vi) determine critical control points; and (vii) establish critical limits for each critical control point.

### Team

The international and multidisciplinary nature of the problems posed by Ebola virus meant that we – i.e. the members of the research team – were obliged to conduct our analysis via a mixture of face-to-face meetings and email exchanges. The research team included experts in disaster response, environmental health, Hazard Analysis of Critical Control Points protocols, infection control nursing, infectious disease epidemiology, public health, risk assessment, small water systems, virology and water and sanitation engineering. The team members were drawn from 19 different institutional departments spread across multiple countries within Africa, Europe and North America. Our analysis began when team members from the University of East Anglia (Norwich, England) held a series of small meetings. The progress made in these meetings was then shared with a wider group of team members – for comment and feedback – before a two-day face-to-face meeting in Nairobi, Kenya, that was attended by all of the team members from Africa and several of those from Europe.

### Process

In the analysis, a systematic approach that allows for the synthesis of expert opinion is combined with the available evidence. This can bring clarity in an otherwise complex public health system. In our early meetings we concentrated on defining the most important waste products – in terms of risk of transmission – and then creating initial flow diagrams representing the pathways that could be used for the collection and disposal of each of these waste products. The diagrams were then shared with other team members, by email – so that a wider group of experts could comment on them – before they were reviewed and simplified in a face-to-face meeting. At the two-day meeting, experts from the fields of health interventions, sanitation and wastewater hygiene gave their views on the flow diagrams. This review and a final critical analysis by an international panel of experts led to further modifications to – and simplifications of – the diagrams.

We considered a hazard to be a process – within a developing world setting – that could lead to human contact with waste material contaminated with Ebola virus and so provide the opportunity for transmission of the virus to another person. Taking into account the likely viral load of the contaminated material and based on the frequency with which each hazard was likely to occur, we grouped the hazards into high-, medium- and low-risk categories.

Following the validation of each flow diagram, the research team determined appropriate critical control points – i.e. the points at which there is an opportunity to reduce or eliminate the risks of virus transmission. The team then made so-called critical limits, for each identified critical control point, using a combination of expert knowledge – followed by validation via examination of the relevant published data on the epidemiology, prevention and control of Ebola virus disease – and the current relevant recommendations of Médecins Sans Frontières and the World Health Organization (WHO).

### Recommendations

We used the results of the analysis to develop recommendations for the management of waste produced in the care of cases of Ebola virus disease.

## Results

### Hazard analysis

Our assessment of waste disposal within developing world settings affected by Ebola virus disease revealed multiple hazardous practices linked to the collection, transportation, disposal, cleaning and storage of waste (Table 1). We believe that, if managed poorly, each of these practices presents an unacceptable level of risk of the transmission of Ebola virus. All the practices that we categorized as high-risk involved potential contamination with blood: the collection, transportation, cleaning and shared use of blood-soiled fomites and the shared use of latrines contaminated with blood or bloodied faeces (Table 1). The practices identified as medium-risk were the collection and transportation of material contaminated with bodily fluids other than blood, the shared use of latrines soiled with such fluids, the cleaning and shared use of fomites soiled with such fluids and the contamination of the environment during the collection and transportation of blood-contaminated waste (Table 1). All of the other activities and practices linked to waste disposal were deemed to present a lower level of risk of the transmission of the virus.

**Table 1:** Summary of the Hazard Analysis of Critical Control Point (HACCP) assessment for the disposal of waste potentially contaminated with Ebola virus

Potential hazard by critical control point	Level of concern about contaminated materials		Recommendations^b^
	with blood	with bodily fluids other than blood^a^	
**1. Latrine use**			
Contamination of environment	High	Medium	– Suspected and confirmed cases use isolated and segregated latrines and keep pit secure for 7 days^10,24^ after last use by suspected case. – Avoid surface water inflow by using external channels or concrete surroundings, and ensure adequate quality of construction to limit risk of collapse and contamination of groundwater sources.^17^ – Using a single-use cloth – which should subsequently be incinerated – clean surfaces with water and detergent. Then wipe 0.5% chlorine solution^16^^,18–21^ over all surfaces, including door handles, toilet seat, floor and walls.^7^ – Wash hands with soap and water after using latrine.
**2. Washing and cleaning**			
Contamination of cleaner	High	Medium	– Provide proper training of cleaners and ensure experienced supervision. – Use water and detergent for cleaning, followed by 0.5% chlorinated water for disinfecting.^10,18,19^ – Treat wastewater as per CCP12.
**3. Reuse or shared use of fomite**			
Inadequate cleaning	High	Medium	– Avoid reuse where possible and dispose as per CCP8. If reuse is essential, wear full PPE when washing reusable materials or products.^c^ – Check fomite for damage and suitability for reuse. If reuse is possible, clean fomite using a moist single-use cloth, which should then be incinerated. Following cleaning, if possible, with a wash with water at > 60 °C.^16^^,22^ If not possible, soak in 0.5% chlorine solution^18,19^ for a minimum of 30 min, after removing most organic material,^ 10,^^16^^,21,22^ and then let air-dry before transporting for reuse.
**4. Transport**			
Splashing on handler	High	Medium	– Avoid handling fresh waste. If unavoidable, wear full PPE and employ appropriate hand hygiene measures.^c^
Contamination of vehicles and/or containers	High	Medium	– At end of each transportation or shift, using a moist single-use cloth that should subsequently be incinerated, clean vehicles and containers with water and detergent. Following cleaning, disinfect using 0.5% chlorine solution.^16^^,18,19,21^ If cloth must be reused, wash with warm water and detergent while wearing appropriate PPE to remove organic matter. Then soak in 0.5% chlorine solution for a minimum of 30 min and rinse with cold water.^ 23^ – Always wear full PPE when cleaning vehicles and containers and disinfect or burn PPE after use.
Contamination of environment	Medium	Low	– Use leak-proof containers – e.g. plastic barrels with secure lids – for contaminated items.^17^ – Using a single-use cloth that should subsequently be incinerated, clean outer surfaces of vehicles and containers before and after use with water and detergent. If cloth must be reused, wash with warm water and detergent, while wearing appropriate PPE, to remove organic matter. Then soak in 0.5% chlorine solution for a minimum of 30 min and rinse with cold water.^23^ Following cleaning, disinfect using 0.5% chlorine solution.^ 10,18,19,21^ – Enclose and/or isolate site. – Spills should be covered first with a cloth, to avoid splashing or dispersion of fluids. Then wipe up spill with rags and dispose of rags through incineration. Clean the area with a detergent and water and then disinfect by wiping with 0.5% chlorine solution.^ 18,19^
**5. Disposal of sharps**			
Contamination of handler	High	Low	– Sharps should be segregated from other waste at point of generation,^ 17,21,23^ placed in puncture-resistant, sealed biohazard-labelled containers and disposed of appropriately, as local facilities allow.^ 17,23^
**6. Emptying of latrine when more than two thirds full**			
Contamination of handler	Variable, depending on age of waste, construction of latrine etc.	Variable, depending on age of waste, construction of latrine etc.	– Wait a minimum of 7 days after last use by a known case before desludging.^ 19,24^ – If not possible to wait 7 days, wear full PPE.^ 25–27^^,b^
**7. Storage**			
Exposure to contaminated waste	Variable, depending on age of waste	Variable, depending on age of waste	– Segregate waste into a secure nonporous container and destroy within 24 h.^19^
**8. Burning of waste**			
Incomplete combustion	Low	Low	– If waste is to be burned, use an incinerator – that reaches sufficient complete burning temperatures and meets environmental emission standards – according to manufacturer’s operating manual. If an incinerator is not available, burn in a barrel or pit with sufficient additional combustible material to ensure complete combustion.^19^ – If large volumes of waste need to be burned, divide into smaller volumes before burning.^19^ – PPE should be worn but extreme caution needs to be taken to avoid the handler’s PPE catching alight.
**9. Cleaning and disinfecting of non-human waste**			
Theft and reuse	Low	Low	– For fabric waste – e.g. bed linen and clothing – discard if possible. If reuse necessary, wash with warm water and detergent, while wearing appropriate PPE, to remove organic matter. Then soak in 0.5% chlorine solution for a minimum of 30 min and rinse with cold water. – For hard waste – e.g. crockery and buckets – wash with a detergent, while wearing appropriate PPE, to remove organic matter. Then soak in 0.5% chlorine solution for a minimum of 10 min and rinse with cold water. – Items can be reused if not damaged. For items not suitable for reuse, dump in a secure area and limit animal access to the secure area.^ 10,^^16^^,18,19,21^
**10. Burial of waste**			
Digging up or theft of health-care waste	Low	Low	– Bury in reliably secure areas, with limited access to animals, and keep secure for 14 days after last disposal. – Acidify or soak in 0.5% chlorine solution for 30 min^10,^^16^^,18,19,21^ before dumping.
**11. Disposal on ground**			
Contamination of food crops	Low	Low^13^	– Prevent disposal onto ground used for food crops and ensure that all crops are handled and prepared according to appropriate food safety guidelines.^28^
Contamination of water supply	Low	Low	– Ensure water supply point is designed to prevent contamination following principles of sanitary assessments included in water safety plans.^12^ – Encourage safe water handling and storage practices and encourage proven household water treatment methods – e.g. filtration, chlorination or boiling.^12,19^
**12. Discharge and treatment of wastewater through sewer**			
Contact of general public with virus via open sewers	Low	Low	– Give public health education to community representatives and construct physical barriers.^12^ Ensure appropriate conditions of carriage – in many places effluent streams are used by neighbours^17^ – by following sanitation safety planning guidelines.^17,29^
Contact of sewage workers with virus	Low	Low	- Ensure standard PPE and hygiene practices are followed.^30^
**13. Open defecation**			
Human and animal contact with virus via human excrement	Low	Low	– Discourage open defecation and encourage pit latrine use. Remove excrement to a pit latrine or bury at a minimum depth of 0.5 m. If unavoidable, dump excrement in secure area.

CCP: critical control point; PPE: personal protective equipment.

^a^ Including urine, faeces and wash water.

^b^ During the execution of this recommendation, appropriate hand hygiene must be employed and full PPE worn, with the correct protocols observed. After each use, PPE should be treated as an infected fomite and either disinfected or burned.

^c^ Due to the nature of Ebola viruses, there must be 100% compliance with this recommendation.

### Control points and limits

We identified 13 critical control points – i.e. 13 points at which there is an opportunity to adopt measures to reduce the risks of transmission. For each such point, following extensive consultation and cross-referencing with the existing literature, one or more potential hazards were identified. Then, one or more sets of recommendations were made for reducing the risk presented by each identified hazard (Table 1). All of the critical control points identified could be assigned to one of five categories: waste collection, waste transportation, waste disposal, waste storage or waste cleaning. The recommendations that we made for each critical control point derive from a combination of basic infection control – e.g. employing thorough hand hygiene measures – Ebola virus-specific recommendations produced following consultation with the existing literature and suggested behaviour changes that could reduce the chances of a person exposing themselves to increased transmission risk.

Several of the recommendations we made include fundamental aspects of infection prevention and control – e.g. the correct and proper use of full personal protective equipment, appropriate hand hygiene and thorough cleaning followed by the use of an appropriate disinfectant.

## Discussion

We used analysis based on the Hazard Analysis of Critical Control Points framework to make recommendations for protecting health workers, other staff in health facilities and the wider public from the risks posed by the disposal of potentially infected liquid and solid waste generated in the care and treatment of patients infected with Ebola virus. During this analysis, 13 critical control points associated with the collection, transportation, disposal, storage and cleaning of waste, the use and emptying of latrines and fomite reuse were identified. Our analysis took account of existing evidence presented in the published literature and guidance published by international organizations. When there were no or few relevant data available, expert opinion from an international panel of experts with expertise in environmental health, epidemiology, health-care provision in low-income countries, virology and water and sanitation engineering was sought.

The framework allows for a rapid identification of the risks associated with a known hazard.^13^ In the present study, that known hazard was the transmission of Ebola virus between humans. The 2014–2016 Ebola virus disease outbreak resulted in widespread localized transmission of the virus. Much of this transmission was attributed to the social, environmental and cultural characteristics of the worst-affected countries,^10^ including inadequate health-care infrastructures that, even before the outbreak, were under great stress as the result of high population densities in urban areas.^31,32^ For Ebola virus disease, multiple gaps still exist in our understanding of potential transmission mechanisms and control measures.^10^ In the context of such knowledge gaps, the framework provides a useful systematic approach that focuses on the prevention of harm and synthesizes hard evidence and expert opinion.^15^ Given such knowledge gaps, however, it remains possible that future research may produce results that require our current recommendations to be modified.

Previous studies have shown that direct contact with a symptomatic person with Ebola virus disease is typically associated with relatively low risk of transmission during the early stages of the disease – whereas the risk of indirect transmission increases as the disease progresses.^ 11,33,34^ The transmission of the virus during burial rituals – which fall outside the scope of the present study – are known to be particularly hazardous practices.^ 11,33,34^ The transport and disposal of potentially contaminated waste also present opportunities for the virus to leave the household or care centre and enter the wider community or environment. In our analysis, taking into account the likely viral load within the contaminated waste and the likely opportunity for transmission of the virus via a particular activity, we only associated activities involving blood-soiled waste with relatively high risks of Ebola virus transmission. For blood-soiled waste, the highest risks were associated with activities that could bring the waste into direct contact with either another person – e.g. the cleaning of a blood-contaminated container – or a potential viral vehicle – e.g. a container used to transport bloodied waste or a contaminated fomite passed to another person without being adequately cleaned. Each of these risks could be eliminated or, at least, substantially decreased through the careful implementation of the recommendations outlined in Table 1. As there is some evidence to suggest that spraying a potentially contaminated surface with disinfectant may promote transmission,^35^ we recommend containment of the spill, followed by cleaning of the surface with water and detergent and finally wiping with 0.5% chlorine solution. This recommendation, which follows published guidelines,^ 18,19,36^ was kept relatively simple because many of those undertaking the cleaning and the preparation of solutions for disinfecting within non-clinical settings would not be able to record times or determine chemical concentrations accurately.

Ebola virus can rarely be detected in the faeces or rectal swabs from persons with Ebola virus disease, even during periods of acute illness,^ 12,37–39^ and little is known about the persistence of the virus within faeces.^11^ With this in mind, we considered faeces to be less of a transmission risk than blood. It has been estimated that approximately one in four samples of non-blood bodily fluids collected during active illness was likely to contain viable virus.^14^ It remains possible that these non-blood bodily fluids only contain Ebola virus when contaminated with non-visible blood.

Whenever fomite-related transmission has been suspected, it has rarely been possible to exclude the possibility of direct human-to-human contact as the transmission route.^9^ We considered the risks of transmission via food crops and via disposal of waste directly onto the ground to be low – primarily because of the lack of evidence of such transmission^40^ but also because solar radiation and other environmental stressors, such as antiviral microbial activity in soils, may rapidly inactivate the virus. However, it is worth noting that there is evidence highlighting contact with raw sewage as a transmission route for many other pathogens.^41^ Previous research into filoviruses has shown that, on clear days at tropical latitudes, it can take as little as 20 min to decrease the infectivity of sun-exposed virus by 90%.^42^ Although some researchers have been concerned about the potential risk to wastewater workers posed by viable Ebola virus in sewer systems,^43^ others believe that any predisposal treatment of sewage that would be potentially contaminated with Ebola virus could increase the transmission risk.^44^ Although some Ebola virus survived more than two days in sterilized wastewater,^45^ the United States Centers for Disease Control and Prevention believes that standard personal protective equipment for sewage workers provides sufficient protection.^30^

In conclusion, our analysis has shown that, while there is limited relevant literature, waste from the care of cases of Ebola virus disease should not be discounted as a potential transmission route. The cleaning, collection, disposal, storage and transportation of waste contaminated with blood probably present the greatest waste-related transmission risks, particularly when the blood has come from a person in the advanced stages of the disease. We believe that waste contaminated with other bodily fluids poses a substantially lower transmission risk, which decreases further with time after contamination. However, it is important to keep in mind that our analysis was mainly based on the 2014–2016 Ebola virus disease outbreak. This outbreak was unusual because it expanded into densely populated urban centres and there was sustained transmission of the virus within such environments. Previous outbreaks of Ebola virus disease have predominantly occurred in small, rural communities where containment and isolation were more feasible. The 2014–2016 outbreak was far larger than any previously recorded outbreak and it is possible that the elevated quantity of virus entering the environment may yet reveal transmission routes that have gone undetected or unnoticed in previous outbreaks. In the meantime, our recommendations should be followed to ensure that the potential for Ebola virus transmission via waste materials is minimized.
